# A double-blind, randomized, and active-controlled phase III study of Herbiron drink in the treatment of iron-deficiency anemia in premenopausal females in Taiwan

**DOI:** 10.3402/fnr.v60.31047

**Published:** 2016-06-23

**Authors:** Ching-Tzu Lee, Cherng-Jye Jeng, Lian-Shung Yeh, Ming-Shyen Yen, Shih-Ming Chen, Chyi-Long Lee, Willie Lin, Chun-Sen Hsu

**Affiliations:** 1 Chinese Medicine Division, Wan Fang Hospital, Taipei Medical University, Taipei, Taiwan; 2 Department of Obstetrics and Gynecology, Kaohsiung Medical University Hospital, Kaohsiung, Taiwan; 3 School of Medicine, College of Medicine, Kaohsiung Medical University, Kaohsiung, Taiwan; 4 Department of Obstetrics and Gynecology, China Medical University Hospital, Taichung, Taiwan; 5 Department of Obstetrics and Gynecology, Taipei Veterans General Hospital, Taipei, Taiwan; 6 Department of Obstetrics and Gynecology, Taiwan Adventist Hospital, Taipei, Taiwan; 7 Department of Obstetrics and Gynecology, Linkou Chang Gung Memorial Hospital, Taoyuan, Taiwan; 8 Microbio Co., Ltd., Taipei, Taiwan; 9 Department of Obstetrics and Gynecology, Wan Fang Hospital, Taipei Medical University, Taipei, Taiwan

**Keywords:** iron-deficiency anemia, herbiron, ferrous bisglycinate chelate, elemental iron, paeoniae radix, premenopausal women

## Abstract

**Background:**

About 468 million non-pregnant women are estimated to suffer from iron-deficiency anemia (IDA) worldwide. The highest prevalence of IDA occurs in the Taiwanese population.

**Objective:**

To evaluate the effectiveness of Herbiron to increase iron absorption in women with IDA.

**Design:**

Phase III double-blind, randomized, active-controlled, and parallel comparative study enrolled 124 patients with IDA and consisted of a 2-week run-in period, randomization, 12 weeks of supplementation, and 4 weeks of follow-up. The treatment group received Herbiron drink 50 mL p.o., b.i.d., before meals (daily iron intake: 21 mg/day) plus placebo tablets. The control group received a ferrous sulfate tablet, t.i.d., plus placebo 50-mL drink before meals (daily iron intake: 195 mg/day).

**Results:**

Both treatments significantly improved hemoglobin and all secondary efficacy endpoints. Most IDA patients treated with Herbiron or ferrous sulfate finished the study in the normal range. Ferrous sulfate treatment induced a rapid rate of hemoglobin synthesis, which plateaued by week 8, whereas Herbiron treatment increased the rate of hemoglobin synthesis more slowly, likely due to its nine-fold lower iron content. Gastrointestinal adverse events (diarrhea, abdominal pain, dyspepsia, and nausea) but not infectious adverse events were significantly more common in the ferrous sulfate group (*n=*11, 18.3%) than those in the Herbiron group (*n=*1, 1.6%) (*p=*0.004).

**Conclusion:**

Twelve weeks of Herbiron treatment delivering 21mg of iron or ferrous sulfate treatment delivering 195 mg of iron induced normal hemoglobin levels in 62 or 91% of non-pregnant women with IDA in Taiwan, respectively, suggesting dose-dependent and bioavailability effects.

Nutritional deficiencies, including iron, reduce the health status of all age groups, from infants to the elderly (1). Although iron deficiency is associated with anemia and inadequate blood synthesis, it also leads to abnormal immune function, higher infection rate (2), inadequate regulation of body temperature, and shorter duration of productive work. Iron supplementation of patients with iron-deficiency anemia (IDA) can improve multiple blood variables including hemoglobin levels, serum ferritin levels, transferrin saturation (3), and physical performance (4) as long as the patients are compliant. Currently, strategies to control iron deficiencies include the fortification of foods with iron, dietary diversification, iron supplementation, and antihelminthic treatment (5). The most common forms of iron supplementation are ferrous sulfate, ferrous fumarate, ferrous gluconate, ferrous glycine sulfate, and iron polysaccharide. Ferrous sulfate, the most commonly used ferrous iron salt, is cost-effective and is considered the standard of care for IDA in most clinics (6, 7). Unfortunately, ferrous sulfate supplementation can induce gastrointestinal upset, which appears to be dose dependent and is a common reason for noncompliance (5, 8). Thus, other formulations are needed.

Ferrochel, a ferrous bisglycinate chelate, is an alternative effective treatment in patients with IDA (9) because it has a higher bioavailability administered with food than ferrous sulfate (9, 10). For example, although pregnant females received a lower dosage of iron in Ferrochel, the measured parameters (e.g. hemoglobin, serum ferritin, and transferrin saturation) had improved significantly more in the Ferrochel-treated group than those in the ferrous sulfate group (11). Ferrous bisglycinate chelate is generally recognized as safe for use as a dietary supplement or a food fortificant by the FDA. Ferrochel has been combined with the following four herbal components in the supplement, which is a 50-mL drink manufactured by MICROBIO CO. LTD., Herbiron: Angelica sinensis (Oliv) Diels (Dang Quai) (12, 13), Rehmannia glutinosa (Soe Dee Huang) (14), Rhizoma Ligustici Chuanxiong (Tsuan Chyong), and Paeonia radix Pall (14), which appear to improve the synthesis of erythrocytes and hemoglobin by various mechanisms. Over 8 million bottles of Herbiron have been consumed as a functional food in Taiwan during the past 2 years, and no safety issues have been reported.

The highest prevalence of IDA in the Taiwanese population occur in females of 30 to 50 years old, according to the Nutrition and Health Survey in Taiwan (NAHSIT) during the period of 1993–1996 (15). We hypothesized that Herbiron provides an effective iron supplementation for premenopausal women with IDA. Here, we investigated and evaluated the clinical efficacy and safety of Herbiron compared with ferrous sulfate for the treatment of IDA in premenopausal females of Taiwanese origin. We monitored changes in hemoglobin, the rate of hemoglobin synthesis, iron supply to tissues (iron bound to transferrin), iron stores in tissues (serum ferritin), and tissue needs for iron (serum transferrin receptors) to assess the efficacy of the two iron supplements (16).

## Methods and patients

### Patients

**Fig. 1:**
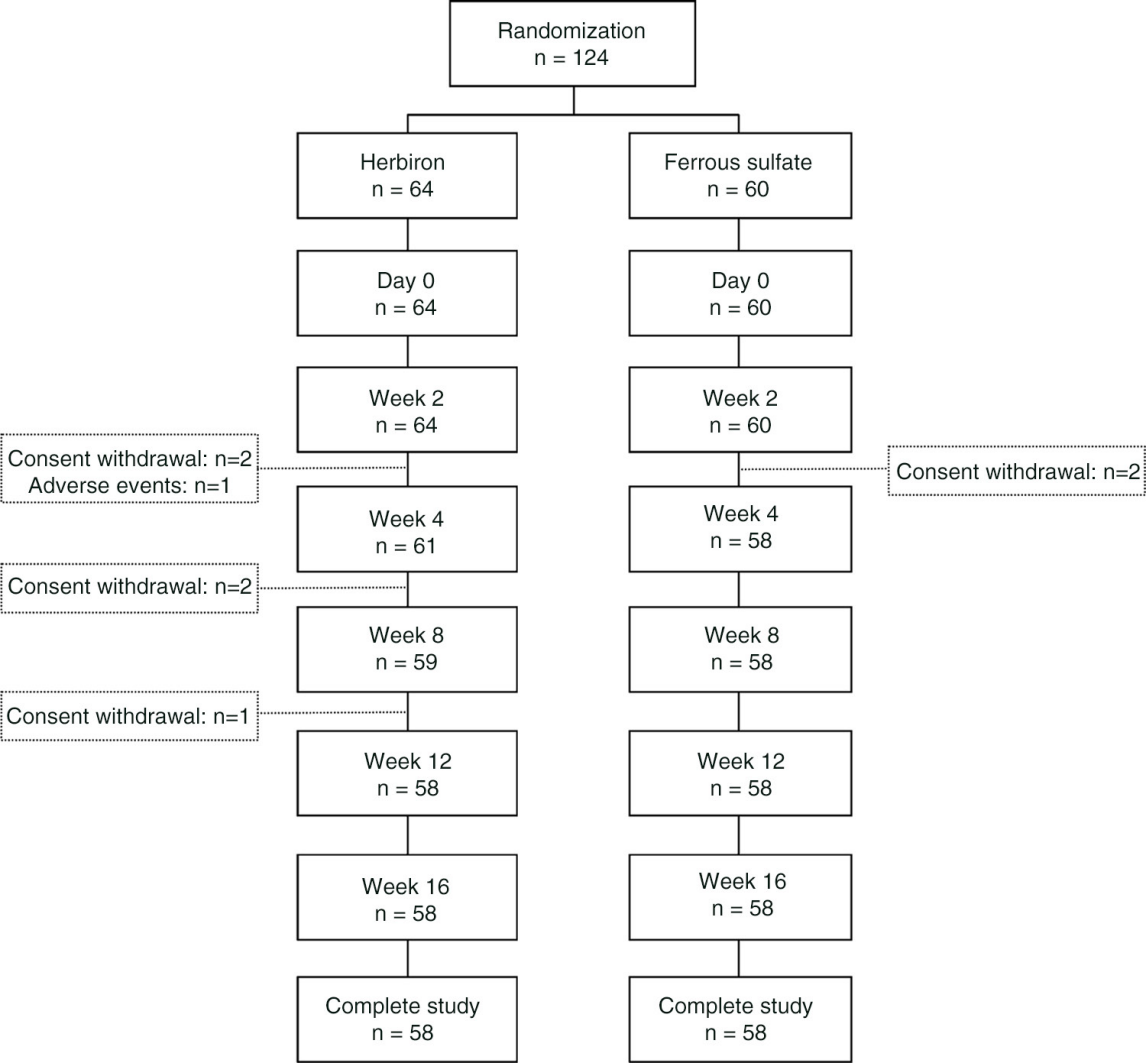
Flow chart of patient disposition.

Consecutive premenopausal women diagnosed with IDA between September 9, 2009, to March 21, 2011, at the Wan Fang Hospital; Taipei Medical University Hospital; Taiwan Adventist Hospital; Taipei Veterans General Hospital; Chang Gung Memorial Hospital, LinKou; or China Medical University Hospital were invited to enroll in the study. A flow chart of patient disposition is shown in Fig. 1. Inclusion criteria were age 12 years or older; hemoglobin between 8 and 12 g/dL, and serum ferritin <12 µg/L; negative serum pregnancy test; and the use of highly effective contraceptive methods for women of child-bearing potential. Menorrhagia defined as excess bleeding during menstruation was allowed. Exclusion criteria were intolerance to oral iron supplementation; intestinal malabsorption; current gastric/duodenal ulcer or gastrointestinal bleeding within 3 months; diagnosis of gastrointestinal malignancy or uterine carcinoma; anemia associated with chronic abnormal uterine bleeding (i.e. irregular periods of bleeding outside of menstruation); history of thalassemia; clinically significant hepatic or renal diseases, which are judged by the investigator to potentially increase a patient's risk; any serious disease that could affect patient safety or study outcome as judged by the investigator (e.g. patients undergoing chemotherapy). The Department of Health, Executive Yuan, Taiwan, and the Institutional Review Boards (IRBs) of all participating hospitals had approved the protocol and informed consent was collected from each participating subject.

#### Two-week run-in period (between screening and baseline visit)

After screening, patients terminated all prior treatments that were related to IDA, including vitamin B12, C, or folic acid supplementation; over-the-counter medications of Chinese herbal formulas if indicated by investigators; any type of iron supplements (e.g. iron-dextran i.v. or i.m.); steroids; and tetracyclines, quinolones, antacids, or other medications known to complex with irons. However, patients were permitted routinely used medications or treatments for other indications, which were judged by the investigator as necessary and did not affect the evaluation of the efficacy and safety of the use of Herbiron drink plus placebo tablets and /or ferrous sulfate plus placebo drink. Relevant information of prior prohibited treatments and concurrent therapy was recorded on the patient's case report form (CRF), including the name of the procedure or drug (generic name), dose regimen, and duration of treatment.

### Study design

After the 2-week run-in period, this phase III double-blind, randomized, active-controlled, and parallel comparative study was carried out for a 12-week treatment period and 4-week follow-up period in accordance with Good Clinical Practice (GCP) guidelines. Treatments were avoided during menstrual periods and restarted after completion of menstrual period. Patients were randomized in balanced blocks per center with an equal probability of receiving the herbal or control treatment (1:1 ratio). The treatment group received Herbiron drink 50 mL p.o., b.i.d., before meals plus placebo tablets, t.i.d, and Herbiron provided a daily iron intake of 21 mg/day. The control group received ferrous sulfate tablets (324 mg, containing 65 mg elemental iron), t.i.d. plus placebo drink, 50 mL p.o., b.i.d. before meals, and the three ferrous sulfate tablets provided a daily iron intake of 195 mg/day, which is similar to the 200-mg elemental iron supplement provided in the recent guidelines (7). The primary objective was to evaluate the efficacy of the two treatments by comparing the change in hemoglobin level (g/dL) from baseline to study end, and the secondary efficacy endpoints including mean corpuscular volume (MCV) difference, serum ferritin ratio, serum iron ratio, total iron-binding capacity (TIBC) ratio, transferrin/serum ferritin ratio (TF-R), hematocrit difference, pain intensity levels (VAS) difference, and RBC distribution width (RDW) difference. The secondary objective was to evaluate the safety between the two methods. The study period for each subject was 18 weeks (2 weeks for the run-in period; 12 weeks for treatment; 4 weeks for follow-up). Subjects were required to make a total of seven visits.

### Statistical analysis

#### Sample size calculation and population for analysis

Sample size calculation was performed using test for non-inferiority based on the difference (Herbiron drink minus ferrous sulfate tablet) in the primary efficacy variable. The variables were set as tolerance margin for non-inferiority at 45%, one-sided statistical significance level at 2.5% (one-tailed *α=*0.025), and 80% power. With common standard deviation at 12 g/dL and the value of limit of non-inferiority computed at –6.5 g/dL, a group size of 55 subjects per treatment group was calculated to demonstrate the non-inferiority of the Herbiron drink to the Ferrous sulfate tablet. The Intention-to-Treat (ITT) population was defined as all randomized subjects who received at least one dose of treatment or control and who had at least one post-baseline assessment for the primary efficacy variable, regardless of their compliance with the protocol. If needed, a Last Observation Carried Forward (LOCF) method was used. Safety population was defined as all randomized subjects who received at least one dose.

#### Data analysis

Demographic and anthropometrics data were expressed as mean and standard deviation. Medical history and pre-existing conditions were shown as number and percentage. The baseline characteristics in all treatment and control groups were compared by Fisher exact test for categorical variables and t-test for continuous variables. Comparisons of differences in primary and secondary outcomes between the two treatment groups were analyzed by generalized linear model (10) with identity link for continuous outcome (e.g. mean change) and logit link for binary outcome (e.g. rate). Baseline values (i.e. study center, age, and body mass index) were considered as covariate in the model. Fisher's exact test was used to compare the incidence of adverse events between two groups. All statistical analyses were performed with SAS software version 9.2 (SAS Institute Inc., Cary, NC, USA). A two-tailed *P<*0.05 indicated statistical significance.

## Results

### Baseline characteristics

A total of 124 subjects were recruited from six study centers and were randomized into two groups: 64 in Herbiron group and 60 in ferrous sulfate group. All randomized subjects received at least one dose of study drug. The two treatment groups were well balanced and showed no significant differences in demographic characteristics, physical attributes, or prevalence of any concurrent disease (Table 1). The mean baseline of hemoglobin of the two groups showed much variation, overlapped, and no significant difference (Table 1). During the study, five subjects in the Herbiron group and two subjects in ferrous sulfate group discontinued due to consent withdrawal. Only one Herbiron-treated patient terminated the study due to an adverse event (moderate diarrhea).

**Table 1:** Comparisons for the baseline characteristics between the two treatment groups

Variable	Herbiron (*n=*64)	Ferrous sulfate (*n=*60)	*p*^a^
Demographics^b^			
Age (years)	41.5±7.31	41.4±7.44	0.96
Weight (kg)	58.5±8.31	59.6±9.64	0.50
Height (cm)	158.6±5.28	159.7±5.52	0.29
Body mass index (kg/m^2^)	23.2±2.88	23.4±3.58	0.80
Primary and secondary endpoints			
Hemoglobin (g/dL)^b^	9.81±1.02	9.85±1.08	0.85
Normal hemoglobin^c^	4 (6.3%)	3 (5.0%)	1.00
Mean corpuscular volume (µm^3^)^b^	72.81±7.39	73.43±8.23	0.66
Serum ferritin level (ng/mL)^b^	4.85±1.72	5.02±1.42	0.67
Serum iron level (µg/dL)^b^	22.08±1.82	21.47±1.78	0.79
Total iron-binding capacity (µg/dL)^b^	426.68±1.36	429.86±1.34	0.89
Transferrin/serum ferritin ratio^b^	5.12±1.45	4.90±1.30	0.46
Hematocrit (%)^b^	30.96±2.61	31.13±2.71	0.73
Pain intensity levels (VAS)^b^	3.30±2.98	3.48±3.18	0.73
RBC distribution width (%)^b^	17.98±3.02	18.07±2.28	0.85
Concurrent disease^c^^,^^d^			
Any	53 (82.8%)	51 (85%)	0.81
Uterine leiomyoma	22 (34.4%)	19 (31.7%)	0.85
Adenomyosis	14 (21.9%)	13 (21.7%)	1.00
Endometriosis	10 (15.6%)	3 (5%)	0.08
Dysmenorrhea	9 (14.1%)	8 (13.3%)	1.00
Menorrhagia	9 (14.1%)	8 (13.3%)	1.00
Menstruation irregular	8 (12.5%)	1 (1.7%)	0.03
Ovarian cyst	5 (7.8%)	5 (8.3%)	1.00
Menstrual disorder	5 (7.8%)	2 (3.3%)	0.44
Endometrial hyperplasia	4 (6.3%)	5 (8.3%)	0.74
Hypertension	4 (6.3%)	2 (3.3%)	0.68
Arrhythmia	4 (6.3%)	0 (0%)	0.12
Uterine polyp	3 (4.7%)	0 (0%)	0.24
Abdominal pain	2 (3.1%)	3 (5%)	0.67
Vulvovaginitis	2 (3.1%)	3 (5%)	0.67
Benign ovarian tumor	2 (3.1%)	3 (5%)	0.67

a Group comparisons were performed by *t*-test for continuous variables and by Fisher's exact test for categorical variables.

b Shown as mean±standard deviation (SD).

c Shown as *n* (%).

d Current diseases with incidence rate >4% were shown.

### Efficacy analysis: comparison of primary and secondary endpoints between groups

**Fig. 2:**
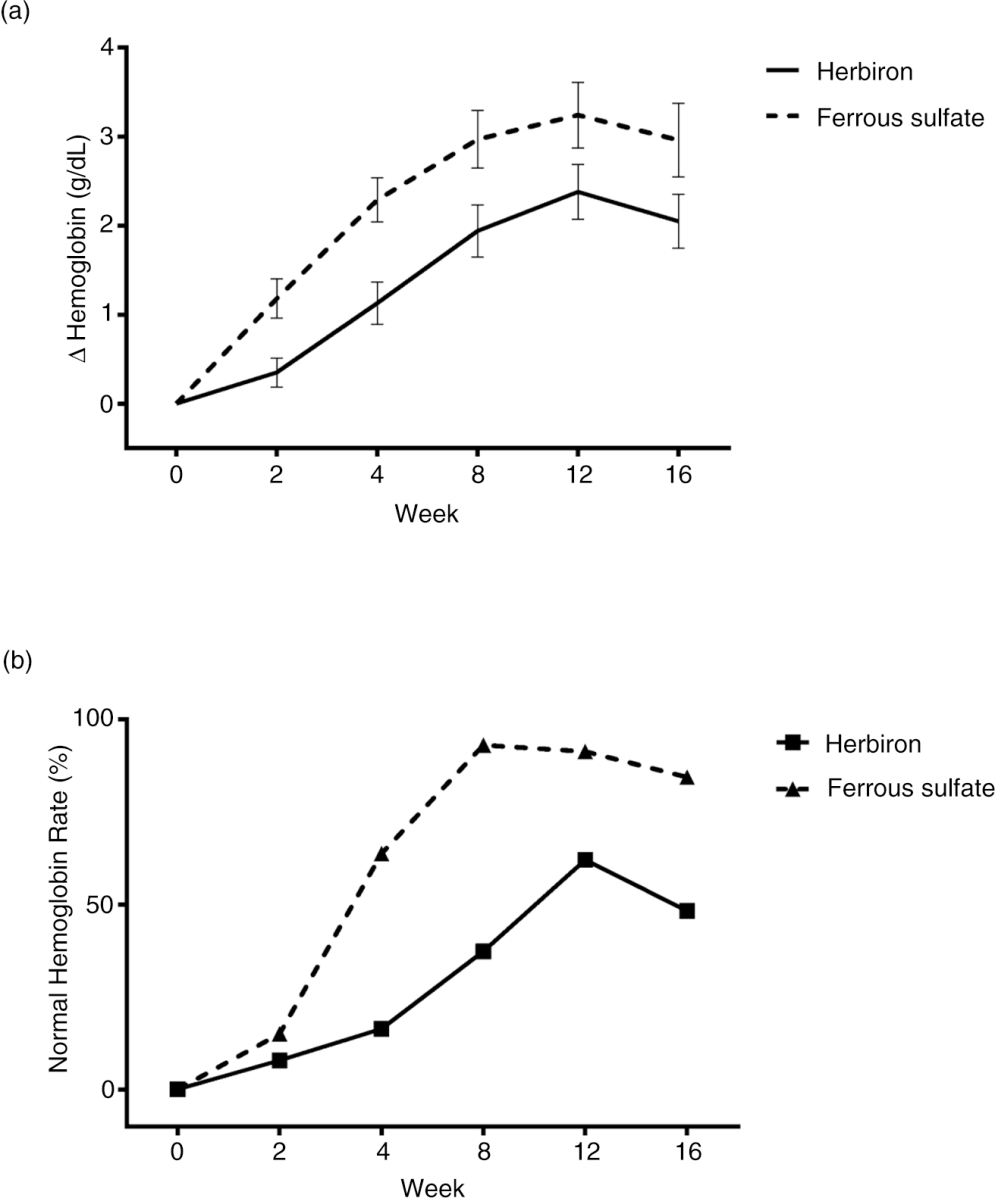
Time course of mean change in hemoglobin level (a) and normal hemoglobin rate (b) during the 12-week treatment and the 4-week follow-up. The multiple-wave assessments were examined by generalized linear model with identity link for hemoglobin level and logit link for normal hemoglobin rate. Mean change±SD.

In this study, 12-weeks of treatment with Herbiron or ferrous sulfate induced a clinically significant improvement in hemoglobin and the majority of secondary efficacy endpoints, including serum ferritin (*p<*0.05). The majority of IDA patients treated with Herbiron (62.07%) or ferrous sulfate (91.38%) finished the study in the normal range. The mean change of hemoglobin level from baseline for the 12-week study was smaller in the Herbiron group than that in the ferrous sulfate group (*p=*0.004 for mean change of hemoglobin) (Fig. 2a). The mean change was continuing to climb at 12 weeks in both groups but the mean change of both groups declined during the follow-up period. Thus, continued supplementation appeared necessary to maintain hemoglobin levels.

Comparison of the rate of hemoglobin synthesis between the two groups (Fig. 2b) suggested that they had different kinetics. The rate of hemoglobin synthesis induced by the ferrous sulfate treatment was rapid between week 2 and week 8, and reached a plateau by week 8 which was maintained to week 12 at the end of the treatment. The rate of hemoglobin synthesis appeared to begin to modestly, but not significantly decline during the follow-up period. In contrast, the rate of hemoglobin synthesis induced by the Herbiron treatment rose more slowly and it had not reached a plateau by week 12. These data raise the possibility that a longer duration of Herbiron treatment may have continued to increase the rate of hemoglobin synthesis. The rate in the Herbiron group appeared to decline more rapidly during the follow-up period than that in the ferrous sulfate group.

The Herbiron treatment group provided 21-mg iron, whereas the control group received 195-mg iron, which is 9.3 times more. Our study design did not compare at equivalent iron supplement dosages as tested in Singh et al. (8). Despite the 9.3 times higher iron content in the ferrous sulfate treatment, Herbiron induced 24.3% at week 12 of the change in hemoglobin as ferrous sulfate treatment, suggesting that dosage and bioavailability factors may affect the clinical results.

**Fig. 3:**
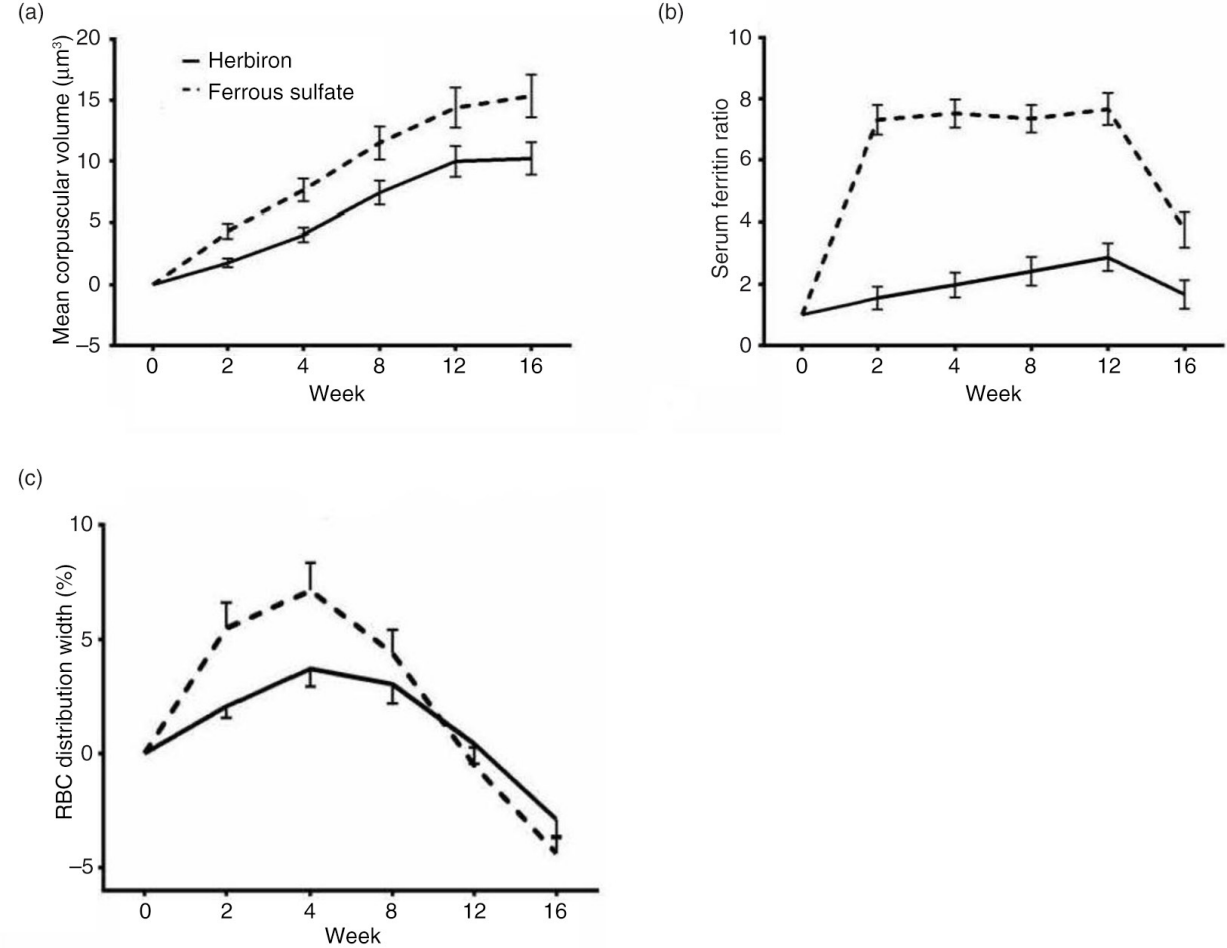
The time course of mean change of secondary outcomes during the 12-week treatment and 4-week follow-up. (a) Mean corpuscular volume; (b) Serum ferritin ratio; and (c) RBC distribution width. The herbiron group (21-mg iron) is depicted by solid lines and the ferrous sulfate group (195-mg iron) is depicted by dashed lines. Mean change±SD.

The mean change in MCV of both groups increased from week 0 to 12 (Fig. 3a). Ferrous sulfate group showed a significantly greater change in MCV than the Herbiron group (*p=*0.02 for MCV). Although the MCV of the ferrous sulfate group continued to rise during the follow-up period, the MCV of the Herbiron group appeared to plateau. Interestingly, the serum ferritin, serum iron, and TF-R of the ferrous sulfate group plateaued at week 2 and maintained the values until the end of the treatment period (week 12) (Fig. 3b and data not shown), whereas these variables showed a slower response in the Herbiron group, with changes evident between week 0 and week 12 (Fig. 3b and data not shown). These data provide further evidence that Herbiron treatment for a longer duration may have continued to show an increase in iron uptake and utilization. No significant differences were found in the mean changes in RDW between the two groups (Fig. 3c).

### Safety analysis

The ferrous sulfate group had a modestly but not significantly higher percentage of people with adverse events (31/60, 51.7%) than the Herbiron group (27/64, 42.2%) (*p=*0.37). All adverse events were either grade I or grade II for both Herbiron and ferrous sulfate groups. Only one person discontinued treatment due to adverse events (diarrhea grade II, Herbiron group). Adverse events occurring in 3% or more of subjects in either treatment group are shown in Table 2. The most frequently reported adverse events of the Herbiron group were upper respiratory tract infection (three subjects, 4.7%), vulvovaginitis (two subjects, 3.1%), vulvovaginal candidiasis (two subjects, 3.1%), irregular menstruation (two subjects, 3.1%), and mitral valve disease (two subjects, 3.1%). Patients in the ferrous sulfate group (*n=*60) suffered most frequently from abdominal pain (four subjects, 6.7%), diarrhea (three subjects, 5.0%), menstrual disorder (three subjects, 5.0%), and cystitis (three subjects, 5.0%). Gastrointestinal adverse events (diarrhea, abdominal pain, dyspepsia, nausea) were significantly more common in the ferrous sulfate group (*n=*11, 18.3%) than those in the Herbiron group (*n=*1, 1.6%) (*p=*0.004). The infection rate (upper respiratory tract infections, vulvovaginitis, vulvovaginal candidiasis, cystitis) was not significantly different between the two groups (Ferrous sulfate group, *n=*8, 13.3%; Herbiron group, *n=*7, 10.9%).

**Table 2:** Comparison of the adverse events between Herbiron and ferrous sulfate groups

Events^a^	Herbiron (*n=*64)			Ferrous sulfate (*n=*60)			
			
	Total	Grade I	Grade II	Total	Grade I	Grade II	*p*^b^
Upper respiratory tract infection	3 (4.7%)	3 (4.7%)	0	2 (3.3%)	2 (3.3%)	0	1.00
Vulvovaginitis	2 (3.1%)	2 (3.1%)	0	2 (3.3%)	2 (3.3%)	0	1.00
Vulvovaginal candidiasis	2 (3.1%)	2 (3.1%)	0	1 (1.7%)	1 (1.7%)	0	1.00
Cystitis	0	0	0	3 (5.0%)	3 (5.0%)	0	0.11
Menstruation irregular	2 (3.1%)	2 (3.1%)	0	1 (1.7%)	1 (1.7%)	0	1.00
Menstrual disorder	1 (1.6%)	1 (1.6%)	0	3 (5.0%)	3 (5.0%)	0	0.35
Mitral valve disease	2 (3.1%)	2 (3.1%)	0	0	0	0	0.50
Dermatitis contact	1 (1.6%)	1 (1.6%)	0	2 (3.3%)	2 (3.3%)	0	0.61
Diarrhea	1 (1.6%)	0	1 (1.6%)	3 (5.0%)	2 (3.3%)	1 (1.7%)	0.35
Abdominal pain	0	0	0	4 (6.7%)	4 (6.7%)	0	0.05
Dyspepsia	0	0	0	2 (3.3%)	2 (3.3%)	0	0.23
Nausea	0	0	0	2 (3.3%)	1 (1.7%)	1 (1.7%)	0.23
Osteoarthritis	1 (1.6%)	1 (1.6%)	0	2 (3.3%)	2 (3.3%)	0	0.61
Back pain	0 (0.0%)	0 (0.0%)	0	2 (3.3%)	2 (3.3%)	0	0.23

a Adverse events with incidence ≥3% either in Herbiron or Ferrous sulfate group were shown.

b Group comparison based on Fisher's exact test.

## Discussion

About 468 million non-pregnant women (approx. 30%) were estimated to suffer from IDA worldwide from 1993 to 2005 (17). The World Health Organization (WHO) has estimated that half of anemia cases are due to iron deficiency (18). Iron deficiency boosts healthcare costs worldwide, hinders learning ability in school children, and reduces adult productivity; although prevention is relatively low cost (5). Ferrous sulfate is the most common iron supplement but it carries a major risk of noncompliance due to side effects (19, 20), most associated with gastrointestinal disturbances (8, 21). Here, we compared the efficacy and safety of a popular herbal formulation in Taiwan, which provides ferrous bisglycinate to the commonly used ferrous sulfate at recommended dosages (7). In this study, 12 weeks of treatment with Herbiron (daily iron content 21 mg) or ferrous sulfate (daily iron content: 195 mg) induced a clinically significant improvement in hemoglobin and all other secondary efficacy endpoints including serum ferritin. Herbiron and ferrous sulfate treatment raised the hemoglobin levels to the normal range in 62.1 and 91.4% of the IDA patients, respectively. Herbiron supplementation steadily increased hemoglobin levels and the rate of hemoglobin synthesis but hemoglobin had not reached a plateau by week 12. The rate of hemoglobin synthesis in the Herbiron group – which provided 9.3-fold less iron – was significantly less than that in the ferrous sulfate group at week 12 and the rate of hemoglobin syntheses declined during the follow-up period. Despite the 9.3-fold lower iron content, Herbiron induced a mean of 24.3% increase in hemoglobin levels by week 12, which was 32.9% of the change induced by a nine-fold higher dose of ferrous sulfate. However, Herbiron treatment (21-mg iron daily) did not meet non-inferiority with the ferrous sulfate group that provided 195-mg elemental iron. Furthermore, these data suggest that the Herbiron group had not yet reached iron saturation and that a longer trial may have further increased iron uptake, utilization, and storage in tissues. Bayraktar et al. (22) has indicated that iron supplementation should continue for 4 to 6 months after the patients reach normal hemoglobin levels to ensure adequate loading of iron tissue stores.

In comparison, four studies showed that ferrous bisglycinate, which is in Herbiron, was non-inferior to ferrous sulfate in synthesis of hemoglobin, and iron-loading of transferrin. Although ferrous bisglycinate supplementation has been shown to provide similar increases in hemoglobin synthesis and transferrin within 6 weeks as ferrous sulfate (120-mg iron) in chemotherapy-treated patients previously (23), the dose of ferrous bisglycinate was 28-mg elemental iron (33% greater than the dose provided in Herbiron) and the dose of ferrous sulfate was 39% lower (23). Second, ferrous bisglycinate at 25 mg was non-inferior to ferrous sulfate (50 mg) in the treatment of pregnant women (24). The dosage of ferrous bisglycinate was 19% higher than in our study and the ferrous sulfate (50 mg) was a 74% smaller dosage (24). Third, ferrous bisglycinate in corn meal showed approximately six-fold higher oral bioavailability than ferrous sulfate in volunteer women (10). Fourth, adult female patients (*n=*375) with low hemoglobin (8–10 g/dL), who received ferrous sulfate (30-mg elemental iron) or ferrous bisglycinate (30-mg iron, combined with 11 mg of elemental zinc, 0.5-mg folic acid, and 7.5-mcg vitamin B12) tablets significantly increased their hemoglobin within 6 weeks (8). In Singh's study with equivalent amounts of elemental iron, the ferrous bisglycinate supplemented group had a significantly greater increase in hemoglobin levels (2 g/dL) than the ferrous sulfate supplemented group (1.65 g/dL) (*p<*0.05) in the 6-week study (8). This ferrous bisglycinate supplement contained 42.9% higher dosage of elemental iron supplement than our study and the ferrous sulfate was an 84.6% smaller dose than provided in our study. Thus, it is conceivable to speculate that the non-inferiority might be attributed to the non-comparable iron intake.

Furthermore, we conducted a systematic literature review with 23 human studies published in PubMed and the data on efficacy and safety of Ferrous sulfate in ID or IDA was analyzed (8, 9, 25–42). Overall, ferrous sulfate of approximately 60-, 120-, and 180-mg elemental iron intake increased hemoglobin in a dose-dependent manner, on average by 1.37, 2.01, and 2.75 g/dL, respectively. The corresponding increases in serum ferritin were 6.15, 13.53, and 14.37 mcg/L. In this current study, 12-week treatment with Herbiron increased hemoglobin by 2.38 g/dL and serum ferritin by 8.9 mcg/L. Our analysis of 26 publications indicate that the effect size from our study drug Herbiron is comparable to one to two tablets of ferrous sulfate per day or 60- to 120-mg elemental iron per day. It is reasonable to speculate that non-inferiority might be met if the comparator was one to two tablets (60-mg to 120-mg iron) of ferrous sulfate. Taken together, these data suggest that Herbiron (21-mg iron) may be non-inferior to a lower dosage of ferrous sulfate.

Ferrous sulfate supplementation is well known for causing gastrointestinal adverse events in a dose-dependent manner (5, 8). Herbiron treatment caused significantly less gastrointestinal adverse events than ferrous sulfate treatment, in agreement with the safety profile of other ferrous bisglycinate formulations (8, 23, 24).

Limitations of the study include differences in the dosage of the elemental iron in the two treatments and the 12-week duration of the study. First, although recent guidelines recommend ferrous sulfate containing 200-mg elemental iron (7), similar to the ferrous sulfate dosage provided in our study, previous reports suggest that lower dosages such as 60-mg elemental iron daily (5) or 15 mg daily (43) may be adequate. Herbiron, which delivers 21-mg elemental iron, increased hemoglobin to the normal range in 12 weeks in most IDA patients. Second, whereas most trials lasted 6 weeks to 60 days, our 12-week trial was not sufficiently long for Herbiron to fill the iron stores in the body in most patients. Thus, the suggestion of Bayraktar et al. that iron supplementation should last an additional 4 to 6 months after hemoglobin is restored to normal range has merit (22).

In conclusion, ferrous sulfate treatment and Herbiron showed clinically significant improvement in hemoglobin from the baseline (*p<*0.001). Herbiron, which provided nine-fold less iron daily than ferrous sulfate, increased hemoglobin and transferrin at a slower rate than ferrous sulfate and may require a longer supplementation schedule. On the contrary, the Herbiron drink induced significantly fewer gastrointestinal adverse events than ferrous sulfate. Taken together, Herbiron warrants further consideration for treatment of iron-deficiency anemia in clinical practice.
